# Permeability changes and effect of chemotherapy in brain adjacent to tumor in an experimental model of metastatic brain tumor from breast cancer

**DOI:** 10.1186/s12885-018-5115-x

**Published:** 2018-12-07

**Authors:** Afroz S. Mohammad, Chris E. Adkins, Neal Shah, Rawaa Aljammal, Jessica I. G. Griffith, Rachel M. Tallman, Katherine L. Jarrell, Paul R. Lockman

**Affiliations:** Department of Pharmaceutical Sciences, West Virginia University Health Sciences Center, School of Pharmacy, 1 Medical Center Drive, Morgantown, West Virginia 26506-9050 USA

**Keywords:** Brain metastases, Fluorescent microscopy, Autoradiography, Astrocytosis, Chemotherapy

## Abstract

**Background:**

Brain tumor vasculature can be significantly compromised and leakier than that of normal brain blood vessels. Little is known if there are vascular permeability alterations in the brain adjacent to tumor (BAT). Changes in BAT permeability may also lead to increased drug permeation in the BAT, which may exert toxicity on cells of the central nervous system. Herein, we studied permeation changes in BAT using quantitative fluorescent microscopy and autoradiography, while the effect of chemotherapy within the BAT region was determined by staining for activated astrocytes.

**Methods:**

Human metastatic breast cancer cells (MDA-MB-231Br) were injected into left ventricle of female NuNu mice. Metastases were allowed to grow for 28 days, after which animals were injected fluorescent tracers Texas Red (625 Da) or Texas Red dextran (3 kDa) or a chemotherapeutic agent ^14^C-paclitaxel. The accumulation of tracers and ^14^C-paclitaxel in BAT were determined by using quantitative fluorescent microscopy and autoradiography respectively. The effect of chemotherapy in BAT was determined by staining for activated astrocytes.

**Results:**

The mean permeability of texas Red (625 Da) within BAT region increased 1.0 to 2.5-fold when compared to normal brain, whereas, Texas Red dextran (3 kDa) demonstrated mean permeability increase ranging from 1.0 to 1.8-fold compared to normal brain. The K_in_ values in the BAT for both Texas Red (625 Da) and Texas Red dextran (3 kDa) were found to be 4.32 ± 0.2 × 10^5^ mL/s/g and 1.6 ± 1.4 × 10^5^ mL/s/g respectively and found to be significantly higher than the normal brain. We also found that there is significant increase in accumulation of ^14^C-Paclitaxel in BAT compared to the normal brain. We also observed animals treated with chemotherapy (paclitaxel (10 mg/kg), erubilin (1.5 mg/kg) and docetaxel (10 mg/kg)) showed activated astrocytes in BAT.

**Conclusions:**

Our data showed increased permeation of fluorescent tracers and ^14^C-paclitaxel in the BAT. This increased permeation lead to elevated levels of activated astrocytes in BAT region in the animals treated with chemotherapy.

## Background

The incidence of metastatic brain tumors in United States is approximately 170,000 patients annually [1]. The most common primary sites for brain metastases are lung, breast, and skin, with more than 70% of the patients account for cancers from lung and breast [2]. The incidence of breast cancer metastases to brain is increasing, as there is a significant improvement in 5-year survival from primary breast cancer [3, 4]. Once diagnosed with metastatic brain tumors from breast cancer, 4 out of 5 patients will die within one year [5].

Conventional chemotherapy fails in metastatic brain tumors due to the presence of blood-brain barrier (BBB)/ blood-tumor barrier (BTB), which prevents a sufficient concentration of chemotherapeutics from reaching lesions [5]. However, we have previously found that there is an increase in drug permeation in metastatic lesions when compared to the normal brain [6, 7]. Many newer strategies to treat metastatic brain tumors include methods to improve chemotherapeutic penetration by overcoming the BBB/BTB, including nanoparticles, osmotic BBB disruption, BBB disruption using ultrasound, etc. [8–11]. All of these strategies have shown increased penetration through BBB, but the effect of chemotherapy on tumor-adjacent healthy tissue has not been thoroughly investigated.

In this study, we hypothesize that the area around tumor is more accessible to drug penetration due to increased vascular permeability and diffusion from the tumor into normal brain tissues, which may result in chemotherapy accumulation and effect in the brain adjacent to tumor (BAT). We tested the penetration of two different fluorescent permeability markers, texas Red free dye (Mol. Wt. 625 Da.) and texas Red dextran 3 kDa. (Mol. wt. 3000 Da.). We then determined the distribution of ^14^C-paclitaxel in normal brain, tumors, and BAT regions. Finally, we studied the effect of chemotherapy on BAT by staining for a marker of neuro-inflammation.

## Methods

### Chemicals & reagents

The fluorescent tracers Texas Red (625 Da) and Texas Red dextran (3 kDa) was purchased from Molecular Probes-Life Technologies (Carlsbad, CA). Dulbecco’s modified eagle medium (DMEM) and Fetal bovine serum (FBS) were purchased from Gibco-Life Technologies (Carlsbad, CA). Cell culture flasks were purchased from Falcon (Corning, NY). Radiolabeled (^14^C)-Paclitaxel (101 mCi/mmol) was purchased from Moravek, Inc. (Brea, CA). Paclitaxel, docetaxel and eribulin was purchased from Selleckchem Chemicals (Houston, TX). Radiolabeled (^14^C)-Paclitaxel (101 mCi/mmol) was purchased from Moravek, Inc. (Brea, CA). Cresyl violet acetate (0.1%) and Cremophore EL was purchased from Sigma-Aldrich (St. Louis, MO). Anti-GFAP antibody (ab4674) was purchased from abcam (Cambridge, MA). All other chemicals and reagents used were of analytical grade and were used as supplied.

### Cell culture

Human MDA-MB-231Br metastatic breast cancer cells were kindly donated as a gift by Dr. Patricia S. Steeg (Canter for Cancer Research, National Cancer Institute, Bethesda, MD). Human MDA-MB-231Br metastatic breast cancer cell line was created from the commercially available MDA-MB-231 cell line by Dr. Patricia Steeg’s lab by repeated cycles of intra-cardiac injection and harvesting from brain metastases in mice [6, 7] . The cells were cultured in DMEM supplemented with 10% FBS. MDA-MB-231Br cell lines were transfected to stably express the enhanced green fluorescent protein (eGFP). All cells used in experimental conditions came from passages 1–10 and were maintained at 37 °C with 5% CO_2_. For all cell preparations for intracardiac injection, cells were harvested at 70% confluency.

### Experimental brain metastases model

All animal handling and procedures were approved by Institutional Animal Care and Use Committee protocol (WVU #13–1207), and all work was conducted following the 1996 NIH Guide for the Care and Use of Laboratory Animals. Human ethics approval and informed consent for this study are not applicable because no human subjects were involved in this study. Female athymic nu/nu mice (24–30 g) were purchased from Charles River Laboratories (Wilmington, MA) and were used for the experimental metastases model in this study. Mice were 6 to 8 weeks of age at the initiation of the brain metastases models and were housed in a barrier facility with chow and water available ad libitum before and after inoculation of tumor cells. For inoculation of MDA-MB-231BR cells, mice were anesthetized under 2% isoflurane and injected with 175,000 cells in the left cardiac ventricle using a sterile 27-gauge tuberculin syringe with the aid of a stereotaxic device (Stoelting, Wood Dale, IL) as previously reported by Adkins et al. [6]. Injection accuracy was evaluated by a pulsatory flash of bright-red blood into the syringe upon little retraction of the plunger prior to injection. After intra-cardiac injection, mice were placed in a warmed (37 °C) sterile cage and vitals monitored until fully recovered. Metastases were allowed to develop until neurologic symptoms like seizures, labored breathing, hunched posture and anorexia appeared (~ 28 days for MDA-MB-231Br), and animals were then anesthetized with ketamine/xylazine (100 mg/kg and 8 mg/kg respectively) prior to Texas Red 625 Da (6 mg/kg in saline) and Texas Red dextran 3 kDa (6 mg/kg in saline) and ^14^C-Paclitaxel (10 μCi/animal, 10 mg/kg in Taxol formulation, Moravek) injection via IV bolus dose (femoral vein). The Texas Red 625 Da (*n* = 6) and Texas Red dextran 3 kDa (n = 6) were allowed to circulate for 10 min prior to euthanasia by decapitation, and ^14^C-Paclitaxel (*n* = 10) was allowed to circulate for 8 h before sacrifice by decapitation. The endpoints for texas red and ^14^C-Paclitaxel circulation times were determined by previous studies [7]. Brains were rapidly removed (less than 60 s), flash-frozen in isopentane (− 65 °C), and stored at negative 20 °C.

### Tissue processing and analysis

**Fig. 1:**
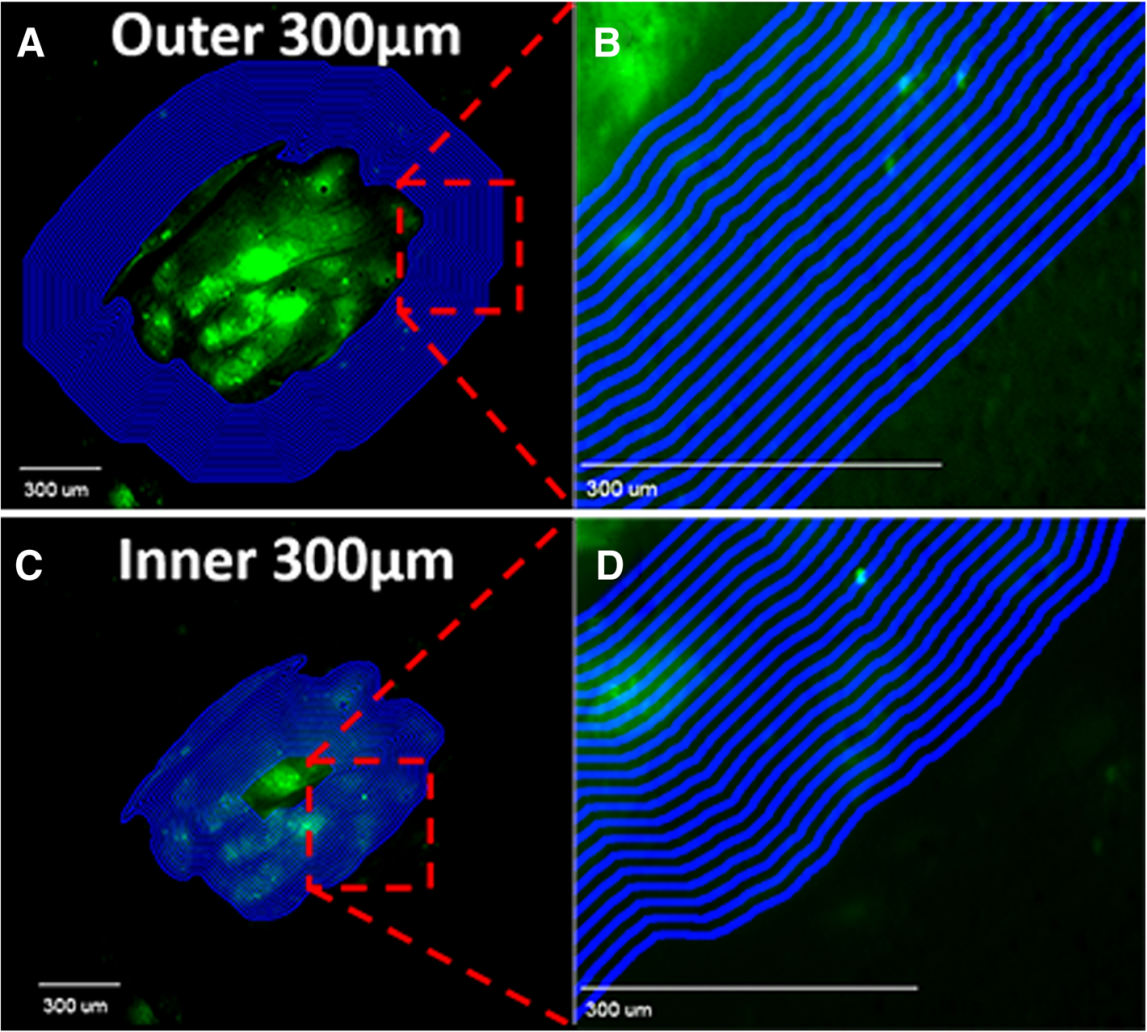
Circumferential Fluorescent Analysis by Quantitative Fluorescence Microscopy. Fluorescent image of eGFP transfected MDA-MB-231Br metastasis in brain with circumferential 8 μm thick regions of interest (ROI) drawn to 300 μm beyond the metastasis margin (**a** and **b**). To distinguish between BAT and tumor regions, the inner 300 μm from the metastasis margin were used to create 8 μm thick circumferential ROIs (**c** and **d**)

Brain slices (20 μm) were acquired with a cryotome (Leica CM3050S; Leica Microsystems, Wetzlar, Germany) and transferred to charged microscope slides. Fluorescent images of brain slices were acquired using a stereomicroscope (Olympus MVX10; Olympus, Center Valley, PA) equipped with a 0.5 NA 2X objective and a monochromatic cooled CCD scientific camera (Retiga 4000R, QIMaging, Surrey, BC, Canada). Texas Red fluorescence was imaged using a DsRed sputter filter (excitation/band λ 545/25 nm, emission/band λ 605/70 nm and dichromatic mirror at λ 565 nm) (Chroma Technologies, Bellows Falls, VT) and enhanced green fluorescent protein (expressed in MDA-MB-231Br) using an ET-GFP sputter filter (excitation/band λ 470/40 nm, emission/band λ 525/50 nm and dichromatic mirror at λ 495 nm) (Chroma Technologies, Bellows Falls, VT). Fluorescent image capture and analysis software (SlideBook 5.0; Intelligent Imaging Innovations Inc., Denver, CO) was used to capture and quantitate images. Binary mask methodology was used to analyze brain slices based upon the eGFP fluorescence from MDA-MB-231Br cells. Binary mask methodology is simply voxel-defined regions of interest where tumor was defined by the presence of eGFP fluorescence from MDA-MB-231Br on a voxel-by-voxel basis. By this methodology, the eGFP fluorescence roughly > 3-fold above background was considered a brain tumor. Once the images were acquired, circumferential fluorescent analysis was performed using software analysis (SlideBook 5.0; Intelligent Imaging Innovations Inc., Denver, CO), where 8-μm thick region of interest (ROI) were drawn 300 μm beyond and within the tumor (Fig. 1a and b). Texas red permeability fold-changes were determined by Texas Red sum intensity (SI) per unit area of metastases relative to that of contralateral normal brain regions. The transfer coefficient (K_in_) of Texas Red tracers were determined in tumor, BAT and normal brain by multiple uptake time approach after analyzing the blood and tumor concentrations of Texas Red tracers as previously described by Mittapalli et al. [12]

.

The unidirectional blood to brain, blood to tumor and blood to BAT transfer constant K_*in*_ was determined for fluorescent tracers using single-time uptake approach [13–15]. A single-time uptake method was used to calculate K_*in*_ because of heterogeneity of the metastatic tumors. K_*in*_ was calculated using the following equation [12, 15]

$$ {\mathrm{K}}_{in}=\frac{C_{br}(T)}{\int_0^t{C}_{bl}(T) dt} $$

Where, C_br_ is the amount of compound in brain/metastatic tumor/ BAT per unit mass of the tissue at time T and C_bl_ is the blood concentration of the tracer.

For ^14^C-Paclitaxel permeation studies, 20 μm thick brain slices were exposed for 20 days to phosphor screens along with tissue-calibrated standards for quantitative autoradiographic analysis. The phosphor screens were developed using GE Typhoon FLA 7000 and images were processed using MCID software (Imaging Research) and Adobe Photoshop to acquire color-coded drug concentrations (ng/g or μg/g) in regions of interest.

### Effect of drugs on BAT

Female athymic nu/nu mice were inoculated with human MDA-MB-231-Br-Luc cells and allowed to develop metastases. On day 21, the presence of metastases was confirmed using an IVIS bioluminescent imaging system and animals are randomly divided into four treatment groups (*n* = 10/group) and then treated with Vehicle (n = 10, saline), Docetaxel (10 mg/kg I.V, once a week, n = 10), Eribulin (1.5 mg/kg I.P, twice every week, n = 10) and Paclitaxel (10 mg/kg I.V, once a week, n = 10). Docetaxel and Eribulin was dissolved in a vehicle composed of 5% Tween 80 and 5% Ethanol in saline, whereas paclitaxel was dissolved in a vehicle composed of 1:1 blend of Cremophor EL and ethanol was then diluted (nine parts of saline to one part of blend) with normal saline for administration. The treatment regimen was continued until mice showed neurological symptoms, and the then mice were sacrificed and the brains were harvested. The brains were sectioned and stained for glial fibrillary acidic protein (GFAP) for the presence of activated astrocytes in BAT region.

### Data analysis

The unidirectional blood-to-brain, blood-to-tumor and blood-to-BAT transfer constant K_*in*_ differences were compared by one-way ANOVA with multiple comparisons (GraphPad® Prism 6.0, San Diego, CA) and were considered statistically significant at *p* < 0.05. MCID software (Imaging Research Inc., UK) was used to quantify permeation of ^14^C-Paclitaxel in brain metastases, BAT and normal brain.

## Results

### BAT permeability

Regional barrier integrity was evaluated using permeability tracers, Texas Red 625 Da and Texas Red dextran (3 kDa), which fall within the upper-limit molecular weight of most conventional and non-biological chemotherapeutic drugs. The margins of metastases were demarcated based on eGFP fluorescence around cancer cell clusters that were confined within 100 μm of each other, as previously described (8). Once the tumor margin was defined for each metastasis, a series of consecutive circumferential masks (8 μm wide) extending 300 μm beyond the original metastasis margin were generated automatically using custom written SlideBook 5.0 software scripts (Fig. 1a and b). The additional 200 μm region was drawn to also allow for analysis of brain distant to tumor. Additional circumferential masks (8 μm wide) that extend 300 μm internally from the metastasis margin were created using the software scripts (Fig. 1c and d).

**Fig. 2:**
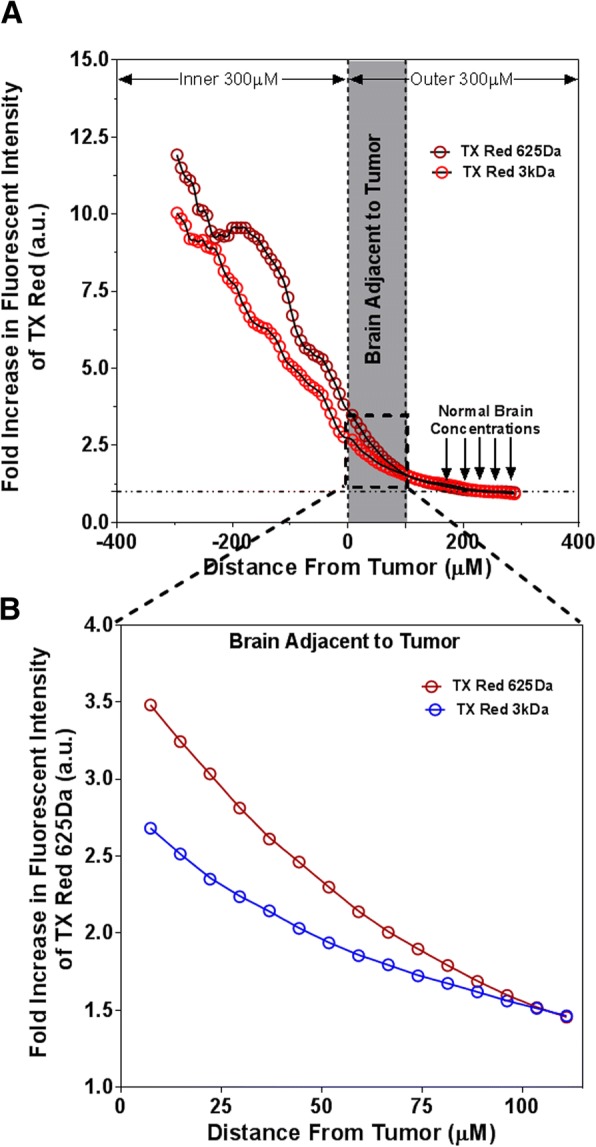
Circumferential fluorescent analysis of Texas Red 625 Da and Texas Red Dextran (3 kDa) in tumor and BAT regions in metastases (**a**). Analysis TR permeation within 100 μm beyond the tumor edge. Fold increase in TR 625da permeability: 1.8–3.8. Fold increase in TRD 3KD permeability: 1–2.5 (**b**)

Texas Red 625 Da and Texas Red Dextran 3 kDa permeation were plotted relative to the distance from the tumor edge for different metastases exhibiting different magnitudes of mean permeability increases (Fig. 2a). Analysis of Texas Red 3 kDa permeation within the BAT region 100 μm beyond the tumor edge for each metastasis demonstrated mean permeability increase ranging from 1.0 to 1.8-fold compared to normal brain (Fig. 2b). The mean permeability of Texas Red 625 Da within BAT region increased 1.0 to 2.5-fold when compared to normal brain.

**Fig. 3:**
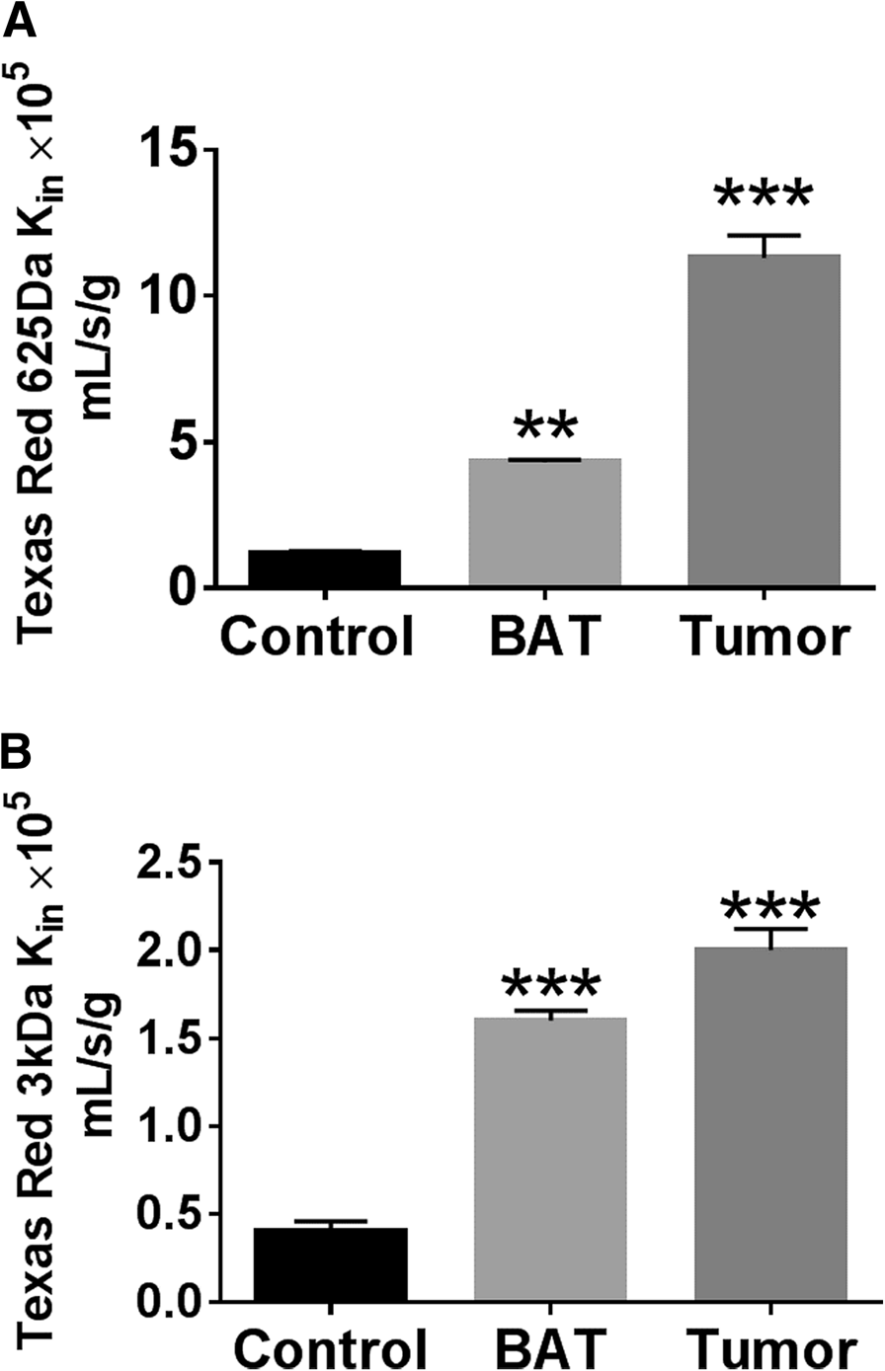
Blood-to- brain transfer coefficients (K_in_) for Texas Red (625 Da) in normal brain (Control), BAT and Tumor regions (**a**). Blood-to- brain transfer coefficients (K_in_) for Texas Red Dextran (3 kDa) in normal brain (Control), BAT and Tumor regions (**b**). The K_in_ was determined by single-time uptake approach. **, *P* < 0.01; ***, *P* < 0.001, respectively. All the data represented here are mean ± SEM; *n* = 6 for all data points

We then calculated K_in_ for tumor, normal brain, and BAT, and we found that there was a significant increase in K_in_ in BAT for both Texas Red free dye and Texas Red Dextran 3 kDa when compared with normal brain (Fig. 3a and b). The K_in_ values for Texas Red 625 Da in normal brain was found to be 1.2 ± 0.16 × 10^5^ mL/s/g. For tumor, it was 11.3 ± 1.9 × 10^5^ mL/s/g, and for BAT the K_in_ was 4.32 ± 0.2 × 10^5^ mL/s/g. The K_in_ values for Texas Red 3 kDa was found to be 0.4 ± 0.14 × 10^5^ mL/s/g, 2 ± 0.3 × 10^5^ mL/s/g and 1.6 ± 1.4 × 10^5^ mL/s/g for normal brain, tumor and BAT respectively.

### Distribution of paclitaxel in normal brain, BAT and tumor

**Fig. 4:**
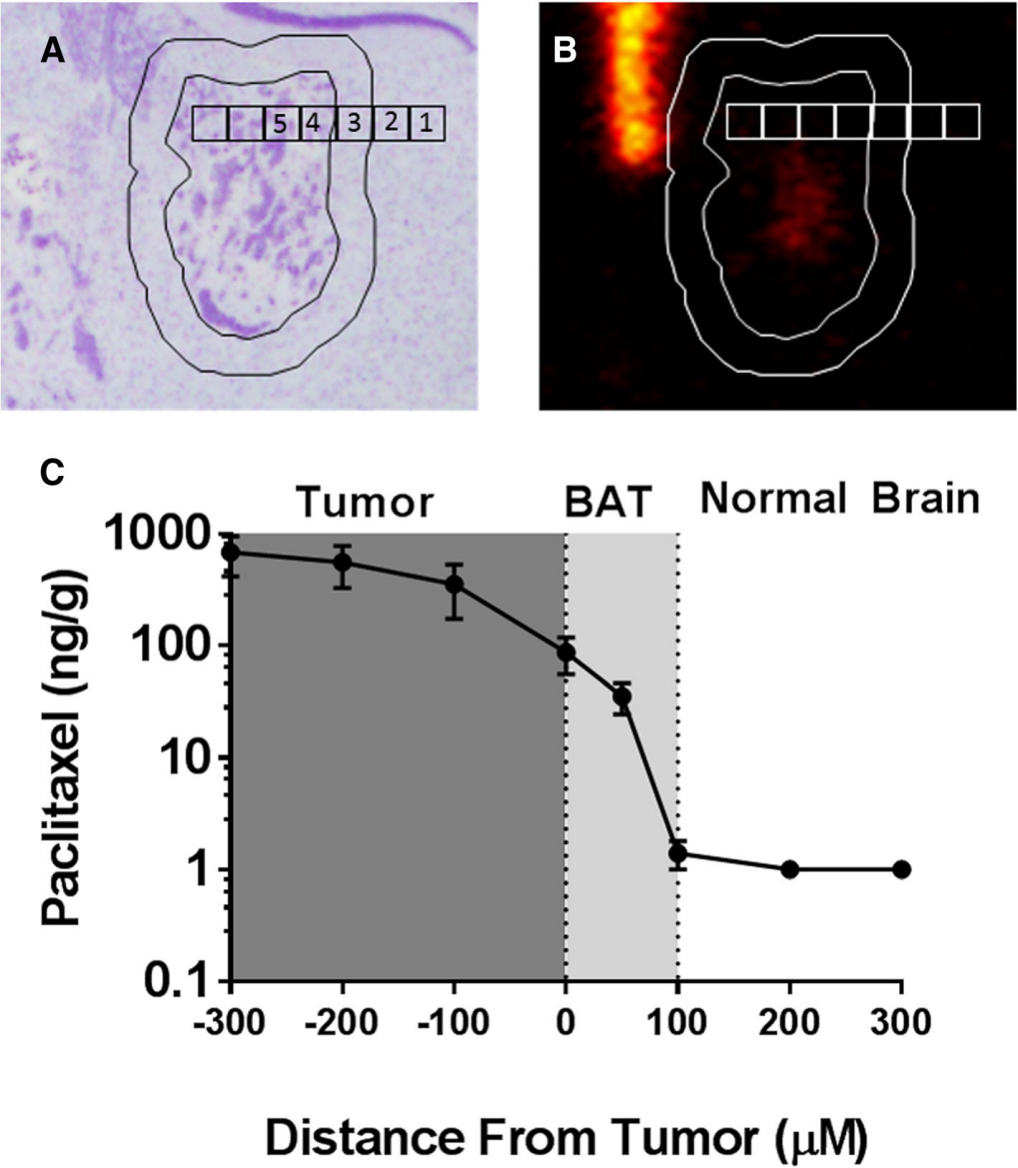
Representative image of 231Br brain metastases (**a**) and corresponding ^14^C-Paclitaxel accumulation (**b**) in metastases 8 h after intravenous administration of radiolabeled paclitaxel. Paclitaxel concentrations from 100 μm squares as shown in image A and B were determined (**1** = 1 ng/g, **2** = 1 ng/g, **3** = 10.5 ng/g, **4** = 293 ng/g, **5** = 261 ng/g). (**c**) Analysis of ^14^C-Paclitaxel concentration in tumor regions (− 300 μm to 0) and normal brain regions (0 to 300 μm). Data are mean ± SEM; *n* = 15 for all data points

After analyzing Texas Red tracer permeability and transfer coefficients in the BAT, we determined the distribution of ^14^C-Paclitaxel using autoradiography. The tumor was identified by cresyl violet stain (Fig. 4a) and the corresponding overlaid autoradiogram (Fig. 4b) was used to analyze the concentrations of paclitaxel in 100 × 100 μm squares (50 × 50 μm squares in BAT) as shown in Fig. 4a and b. We found that there is increase in the concentration of ^14^C-Paclitaxel in BAT regions and the increase in concentration was heterogeneous as seen in the metastases. We found that the concentration of ^14^C-paclitaxel in BAT (0–50 μm) to be 86.7 ± 31 ng/g and BAT (50–100 μm) 35.4 ± 11 ng/g (Fig. 4c), whereas the concentrations of ^14^C-Paclitaxel beyond 100 μm of tumor and normal brain was consistently found to be 1 ng/g. The concertation of ^14^C-Paclitaxel in the tumor was 529 ± 223 ng/g consistent with our previous studies [7].

### Chemotherapeutic drugs induce astrocyte activation in BAT

**Fig. 5:**
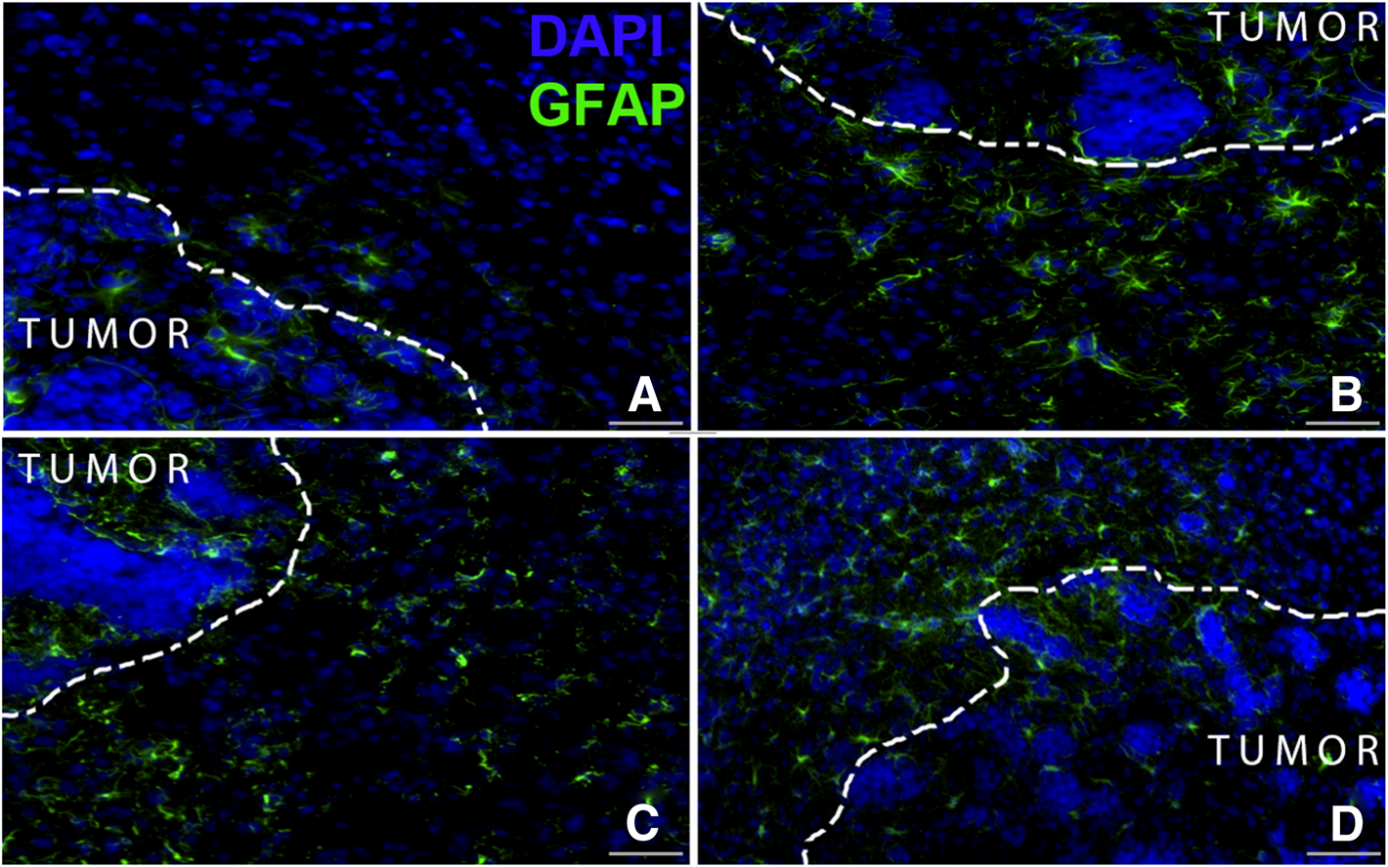
Fluorescent images representing presence of nuclei (DAPI) in blue and activated astrocytes (GFAP) in green after treating with **a**.Saline (Vehicle), **b**. Eribulin (1.5 mg/kg I.P), **c**. Docetaxel: (10 mg/kg I.V), **d**. Paclitaxel: (10 mg/kg I.V). The GFAP expression in BAT regions in chemotherapeutic treated group appears are higher than that of vehicle group. Scale bar = 50 μm

After studying the permeability of tracers and ^14^C-paclitaxel in BAT, we sought to study the effect of chemotherapeutic drugs on BAT. For this study, we treated mice with various chemotherapeutic drugs after the confirmation of metastases as mentioned above. To visualize activated astrocytes, we stained for glial fibrillary acidic protein (GFAP), which is over-expressed when astrocytes are activated [16]. We observed GFAP over-expression in BAT in all the groups treated with chemotherapeutic drugs and found that there is an increase in expression of GFAP in BAT (Fig. 5b-d). However, GFAP expression in BAT in saline treated group was not noticeable (Fig. 5a).

## Discussion

Many studies have shown the permeability and effect of chemotherapy in the brain metastases [7], but surprisingly, there are not many studies investigating those same effects in BAT. With increase in strategies to overcome BBB and BTB to treat metastases [1, 9, 10], it is important to study the permeability in BAT and effect of chemotherapy in metastatic tumors. In this study, we found that the permeability of tracers and ^14^C-palcitaxel increased in BAT when compared to normal brain regions distant to the tumor. We also found that administration of chemotherapeutic drugs induced activation of astrocytes in these adjacent regions.

In this work, we studied permeability for two tracers, Texas red 625 Da and Texas red dextran 3 kDa using quantitative fluorescence microscopy. The methodology was developed based on previous study by Mittapalli et al., [12], where all fluorescent images were captured using the same settings in the microscope to maintain uniformity in fluorescence emission [17]. Permeation of Texas red tracers in brain metastases were previously characterized by Adkins et al. [18], and we found similar fold-increase in tumor core. Unidirectional BBB/BTB transfer constants K_in_ for both dyes were calculated using an established multiple-time uptake approach [13]. The K_in_ values obtained in these studies for normal brain and tumor were consistent with our previous published data [12]. The increased K_in_ values in BAT when compared to normal brain clearly suggest the permeability in BAT region was increased.

Once we had confirmed the increase in permeability of the tracers, we studied the distribution of a chemotherapeutic agent, ^14^C-paclitaxel in BAT. We used quantitative autoradiography (QAR) to determine the distribution of ^14^C-paclitaxel in BAT, normal brain, and within the tumor [19, 20]. We found that there is an increase in accumulation of ^14^C-paclitaxel in the BAT region and this increase is heterogeneous similar to what we have found in brain lesions previously [7]. The increase in permeation of BTB can be accounted for angiogenesis in the tumor [21–23] and the reasons for this heterogeneous permeability within the lesion is due to dynamics of angiogenic process as reported in the previous studies [24]. Also, the vascular endothelial growth factor (VEGF) secreted during tumor angiogenesis disrupt the tight junctions of the BBB which may lead to increased vascular permeability in the BAT [25, 26].

The most common transport mechanism for drugs across BBB is through passive diffusion [27]. For passive diffusion of drugs across the BBB, the drugs which are lipid soluble, low molecular weight (< 400 Da) and which form ≤7 hydrogen bonds are better candidates [28]. Diffusion through lipid membrane like BBB is dependent on molecular volume of the solute, which in turn depends on its molecular weight [29, 30]. BBB permeability decreases 100 fold with the increase is solute’s molecular weight from 300 Da to 450 Da [31]. In addition to solute related limitations, the active efflux transporters like p-glycoprotein (P-gp) and other members of ABC (ATP-binding cassette) family of transporters present at the BBB play a significant role in efflux of chemotherapeutic agents from the brain to blood [32, 33]. However, in metastatic lesions the BBB is disrupted (BTB) which results in an increase in penetration of chemotherapeutic agents [34]. The higher tumor concentration of chemotherapeutic agents in the tumor creates a concentration gradient with the surrounding normal brain allowing the chemotherapeutic agent to diffuse into normal brain [35]. Other studies observed increased blood flow in brain metastases and when compared to normal brain. Regarding permeability, the blood-to-tissue transfer constant (K_i_) for ^14^C-α-aminoisobutyric acid (AIB) was increased in both tumor and BAT when compared to normal brain, suggesting irregular neovascularization with increased permeability in the brain metastases [36–38].

Finally, once we confirmed the increased permeation of tracers and increased distribution of ^14^C-paclitaxel in BAT, we studied the effect of chemotherapy on BAT. After treating with various chemotherapeutic agents, we stained for GFAP to determine whether there was any inflammatory effect of chemotherapeutic drugs in CNS. GFAP is expressed in astrocytes in the brain [39], and when there is injury, inflammation or neurodegeneration in the central nervous system (CNS), the common reaction of astrocytes is hypertrophy, referred to as reactive astrocytosis or activated astrocytes [40–42]. This hypertrophy increases the expression of GFAP in astrocytes as well as the binding affinity to GFAP antibody [43]. Expression of GFAP is altered by many factors like brain injury and disease [16]. Many earlier studies reported the increase in GFAP expression in various diseases such as Alzheimer’s, Amyotrophic lateral sclerosis (ALS), Parkinson’s, Pick’s, Huntington’s and Autism [44–48]. In Autism, increase in autoantibodies of GFAP has also been found in plasma [49, 50]. In the case of acute CNS injuries like brain infarction and traumatic brain injury, there was increase in levels of GFAP in CSF [51, 52]. On the other hand, decrease in GFAP expression was associated with depression and growth of gliomas [53, 54]. We found that treating with chemotherapy, increased the expression of GFAP protein in BAT (Fig. 5), confirming the presence of activated astrocytes after pharmacological chemotherapy regimens.

Recent studies indicate, chemotherapy may induce numerous deleterious effects within CNS such as altered cognitive function, memory and attention [55]. Fading of cognitive function after chronic chemotherapy administration in patients with cancer has been termed “chemo-fog” or “chemo-brain” [56]. With improvements in survival for women with breast cancer over the past decade, there is also increased number of survivors expressing concerns with memory and concentration post treatment [57–59]. Recent studies suggest that the mechanism for chemo-fog is secondary to the toxic effects imposed by sub-lethal concentrations of chemotherapy on the normal cellular population of CNS [60]. Many studies suggests that chemotherapeutic agents not only induce oxidative stress and apoptosis in CNS but they also inhibit proliferation and differentiation of cellular population of CNS leading to abnormal expression of neurotrophic proteins in the brains [61–64].

## Conclusions

In summary, we observed permeation of fluorescent tracers were increased in the BAT compared to normal brain, which was accompanied by increased distribution of ^14^C-paclitaxel_._ This increase in permeation resulted in increased uptake of chemotherapeutic agents and increased the expression of GFAP in regions adjacent to tumor, indicating reactive astrocytosis. As many new clinical strategies to treat brain metastases tend to increase drug permeation, it is also important to study potential damage in normal brain.

## Abbreviations

BAT: Brain adjacent to tumor
BBB: Blood-brain barrier
BDT: Brain distant from tumor
BLI: Bio-luminescence imaging
BTB: Blood-tumor barrier
CNS: Central nervous system
EPR: Enhanced permeation and retention
GFAP: Glial fibrillary acidic protein
P-gp: P-Glycoprotein
QAR: quantitative autoradiography
SEM: Standard error of the mean
